# Heat transfer enhancement in free convection flow of CNTs Maxwell nanofluids with four different types of molecular liquids

**DOI:** 10.1038/s41598-017-01358-3

**Published:** 2017-05-26

**Authors:** Sidra Aman, Ilyas Khan, Zulkhibri Ismail, Mohd Zuki Salleh, Qasem M. Al-Mdallal

**Affiliations:** 1 Futures and Trends Research Group, Faculty of Industrial Science and Technology, Universiti Malaysia Pahang, Lebuhraya Tun Razak, 26300 UMP Kuantan, Pahang Malaysia; 2 Basic Engineering Sciences Department, College of Engineering Majmaah University, Majmaah, 11952, Saudi Arabia; 3 Department of Mathematical Sciences, UAE University, P.O. Box 15551, Al Ain, United Arab Emirates

## Abstract

This article investigates heat transfer enhancement in free convection flow of Maxwell nanofluids with carbon nanotubes (CNTs) over a vertically static plate with constant wall temperature. Two kinds of CNTs i.e. single walls carbon nanotubes (SWCNTs) and multiple walls carbon nanotubes (MWCNTs) are suspended in four different types of base liquids (Kerosene oil, Engine oil, water and ethylene glycol). Kerosene oil-based nanofluids are given a special consideration due to their higher thermal conductivities, unique properties and applications. The problem is modelled in terms of PDE’s with initial and boundary conditions. Some relevant non-dimensional variables are inserted in order to transmute the governing problem into dimensionless form. The resulting problem is solved via Laplace transform technique and exact solutions for velocity, shear stress and temperature are acquired. These solutions are significantly controlled by the variations of parameters including the relaxation time, Prandtl number, Grashof number and nanoparticles volume fraction. Velocity and temperature increases with elevation in Grashof number while Shear stress minimizes with increasing Maxwell parameter. A comparison between SWCNTs and MWCNTs in each case is made. Moreover, a graph showing the comparison amongst four different types of nanofluids for both CNTs is also plotted.

## Introduction

Numerous industrial fluids such as plastics, toothpaste and food stuff are non-Newtonian in nature, therefore study of non-Newtonian fluids have practical significance. To forecast the features of all non-Newtonian fluids, there exists no single model. In non-Newtonian fluids, the relation who connects shear stress and shear rate is non-linear. The constitutive correlation forms equations of non-Newtonian fluids which are higher order and intricate than Navier-Stokes equation. These fluids are hard to handle because of supplementary non-linear terms in the momentum equation. Therefore, a number of mathematical models are presented to predict the behavior of such fluids. Mainly, these models have three types: Differential, rate and integral type models. The differential and rate type are of great importance among them. In differential type fluids stress is deduced by its various higher time derivatives. First grade, second and third grade fluids falls in this category. In rate type, stress and its higher derivatives have an implicit relation. Integral type models describes materials like polymers melts with considerable memory, stress depends upon the history of relative deformation gradient. Rate type fluids have more significance in research as they predict both elastic and memory effects together. Therefore, in the present work, a subdivision of rate type fluids called Maxwell fluid is chosen. This fluid model was initially introduced by Maxwell to predict the elastic and viscous behavior of air^1^. Moreover, Maxwell fluid model is the elementary rate type model used for fluid rheological effects. Maxwell fluid, also known as Maxwell material, has the properties of elasticity and viscosity both. Fetecau and Fetecau^2^ found “a new exact solution for Maxwell fluid flow past an infinite plate”. In another study, Fetecau *et al*.^3^ provided “a note on the second problem of Stokes for Maxwell fluid over an infinite plate oscillating in its plane”. Khan *et al*.^4^ extended Fetecau *et al*.^3^ work by taking into account magnetohydrodynamic (MHD) and porosity effects. Jordan *et al*.^5^ analysed Stokes’ first problem for Maxwell fluids and obtained new exact solutions. Some important studies performed for Maxwell fluid, include the work of Zierep and Fetecau^6^, Sohail *et al*.^7^, Fetecau *et al*.^8^, Jamil *et al*.^9, 10^, Vieru and Rauf^11^, Vieru and Zafar^12^.

However, all these attempts were made for Maxwell fluid for momentum transfer only and the analysis of heat transfer due to convection was not considered. But most of the existing studies are on the convective flow of Maxwell fluid^13–18^ are either solved numerically or analytically by using an approximate method and, exact solutions for such problems are rare. Such investigations are further narrowed down when the convection flow of Maxwell fluid with nanoparticles is considered. According to authors’ knowledge, even not a single article is published in the literature for Maxwell fluid containing nanoparticles where the exact solution is obtained. Such a fluid is known as Maxwell nanofluid or generally a nanofluid.

The idea of nanofluid was initially introduced by Choi^19^, when he dropped solid nano-sized particles in a carrier fluid and the new type of composite fluid was named as nanofluid. Of course the structure of nanofluid is not as simple as of regular fluid, because the suspended nanoparticles can be of different types, shapes and sizes, see for example Aaiza *et al*.^20^, Hussanan *et al*.^21^, Ellahi^22^, Sheikholeslami *et al*.^23–25^ and the references therein. In addition, nanofluid also depends on base fluid. So, the researchers are continuously trying via experimental, theoretical and numerical studies to choose a nanofluid with such a combination of base fluid and nanoparticles that have maximum rate of heat transfer. Amongst various types of base fluid and nanoparticles, in this work we have chosen Kerosene oil as base fluid and carbon nanotubes as nanoparticles. For the sake of comparison, three other types of nanofluids namely engine oil; ethylene glycol and water are also used.

**Figure 1:**
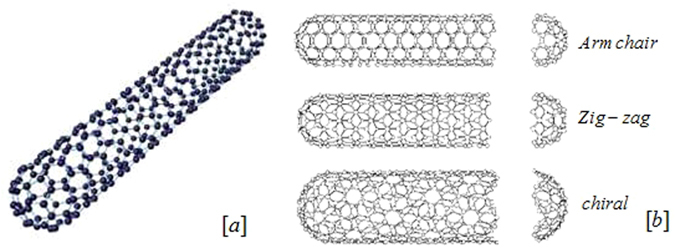
(**a**) Wall structure of SWCNTs. (**b**) Three types of structure of CNTs.

**Figure 2:**
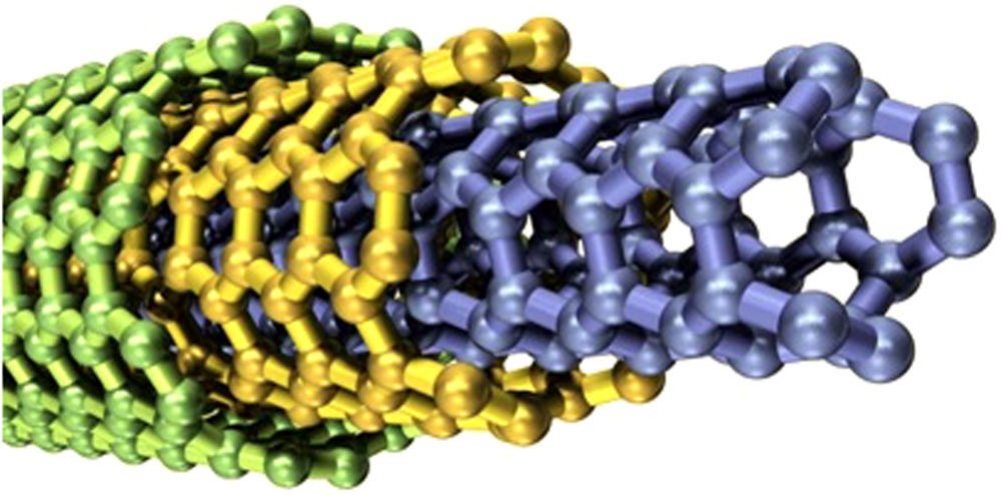
Structure of MWCNTs.

Ramesh and Gireesha^26^ investigated the impact of heat source/sink on a Maxwell nanofluid. Nandy^27^ and Afify and Elgazery^28^ analyzed Flow of Maxwell nanofluid. Cao *et al*.^29^ investigated fractional derivatives of Maxwell viscoelastic nanofluid over a moving plate. Nadeem *et al*.^30^ and Ramesh *et al*.^31^ surveyed Maxwell nanofluids. In all the above studies on Maxwell fluid nanoparticles of tube shapes (CNTs) were not incorporated. Compare to other nanoparticles, CNTs are of great interest for researchers because of their significant thermal conductivities and mechanical strength. They can be bent without any damage; this property makes them useful for high resolution scanning probe microscopy. Two types of CNTs i.e. SWCNTs and MWCNTs are used in this work. Their thermal conductivity is affected by length and diameter which makes them interesting to be arranged in the desirable way in different applications. CNTs are indicated to be the best heat-conducting material. They have applications in fuel cells gas diffusion layers, molecular electronics and current collectors due to their high electrical conductivities. CNTs based filtration devices have been flourished which not only obstruct the mini particles but vanish most bacteria. The wall of SWCNTs consists of one tube of graphite as shown in Fig. 1(a). Further, they can have three types of structures (Armchair, zigzag, chiral) as shown in Fig. 1(b). The graphitic sheet can be rolled in various ways during synthesis, thus there are different types of CNTs. MWCNTs are great choice in nanotechnology as they improve mechanical, electrical and thermal conductivity in the product to which it is added. Their structure is complex because each concentric tube has different diameters and maybe structures as shown in Fig. 2.

Khan *et al*.^32^ studied mixed convection axisymmetric chemically reactive flow of Maxwell fluid. Zhang *et al*.^33^ and Kandasamy *et al*.^34^ examined carbon nanotubes nanofluids. Implementation of Laplace transform for the exact impact of MHD on heat transfer of CNTs nanofluids is studied by Ebaid and Sharif ^35^. Wang *et al*.^36^ detected the impact of CNTs length on their thermal, electrical and mechanical aspects. Competence of CNTs nanofluids as coolants was studied experimentally by Halelfadl^37^. Hussain *et al*.^38^ and Khan *et al*.^39^ analyzed fluid flow with CNTs. Few other attempts on CNTs nanofluids are given in refs 40–44.

The mechanism of convection has three main types which are free, forced and mixed convections. Amongst these three modes of convection, free convection is investigated in this research. In free convection the heat transfer is induced density differences in the fluid owe to temperature gradient. More exactly, free convection is induced by buoyancy force. Free convection has enormous applications in nature and engineering for example microstructure formation, solar ponds, molten metal cooling and much high power output devices^45–48^.

The present work targets to inspect unsteady free convection flow of CNTs Maxwell nanofluid over a stationary vertical plate with constant temperature. Two types of CNTs (SWCNTs and MWCNTs) are suspended in four different kinds of molecular liquids (Kerosene oil, Engine oil, water and ethylene glycol) also known as regular or base fluids. Exact solutions of the problem are evaluated by Laplace transform method. Expressions for velocity, shear stress and temperature are established purely in exact form. The flow and heat transfer features of the present problem are detected for distinct values of non-dimensional parameters. The results thus detected are displayed graphically via computational software MathCAD and discussed in detail.

## Mathematical formulation of the problem

**Figure 3:**
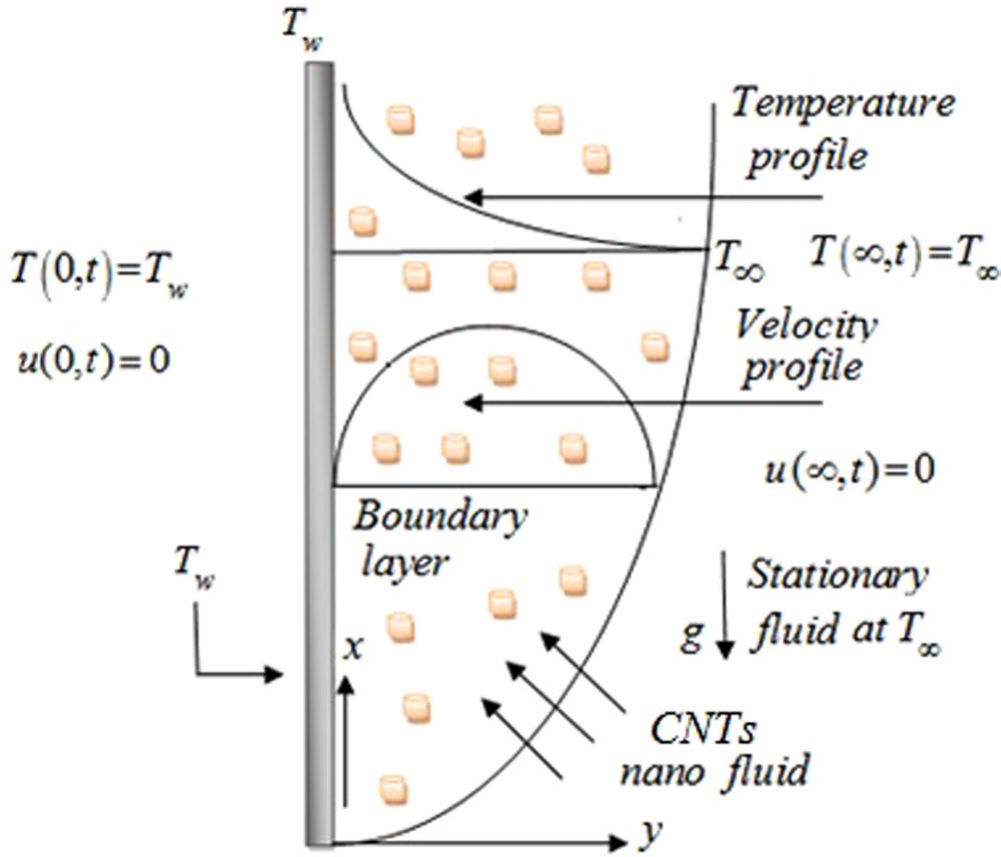
Free convection flow over a hot vertical plate at *T*_*w*_ exposed to plate at *T*_∞_.

Let us consider unsteady flow of an Maxwell fluid with free convection over a vertical flat plate situated in (*x*,*y*)− plane of a Cartesian coordinate system *x*,*y* and *z*. Initially, both the plate and fluid are static with constant wall temperature ${T}_{\infty }^{\ast }$. At time $t={0}^{+}$, the temperature of the plate is raised to a constant value ${T}_{w}^{\ast }$. The temperature approaches to a constant value ${T}_{\infty }^{\ast }$, also known as free stream temperature. The fluid flows due to buoyancy force which is compelled by temperature gradient and there is no external pressure gradient. Kerosene oil-based Maxwell fluid is considered, with two types (SWCNTs and MWCNTs) of Carbon nanotubes added inside it. The physical geometry of the problem is shown in Fig. 3. The equations governing the Maxwell fluid flow related with momentum, shear stress and heat transfer due to free convection are given by the following PDE’s:

1 $${\rho }_{nf}(1+{\lambda }_{1}\frac{\partial }{\partial {t}^{\ast }})\frac{\partial {u}^{\ast }({y}^{\ast },{t}^{\ast })}{\partial {t}^{\ast }}={\mu }_{nf}\frac{{\partial }^{2}{u}^{\ast }({y}^{\ast },{t}^{\ast })}{\partial {y}^{\ast 2}}+(1+{\lambda }_{1}\frac{\partial }{\partial {t}^{\ast }}){(\rho \beta )}_{nf}g({T}^{\ast }-{T}_{\infty }),$$

2 $$(1+{\lambda }_{1}\frac{\partial }{\partial {t}^{\ast }}){\tau }^{\ast }({y}^{\ast },{t}^{\ast })={\mu }_{nf}\frac{\partial {u}^{\ast }({y}^{\ast },{t}^{\ast })}{\partial {y}^{\ast }},$$

3 $${(\rho {c}_{p})}_{nf}\frac{\partial {T}^{\ast }({y}^{\ast },{t}^{\ast })}{\partial {t}^{\ast }}={k}_{nf}\frac{{\partial }^{2}{T}^{\ast }({y}^{\ast },{t}^{\ast })}{\partial {y}^{\ast 2}}.$$

The appropriate initial and boundary conditions are:

4 $$\begin{array}{ll}{u}^{\ast }({y}^{\ast },0)=0;\, & {T}^{\ast }({y}^{\ast },0)={T}_{\infty };\\ {u}^{\ast }(0,{t}^{\ast })=0;\, & {T}^{\ast }(0,{t}^{\ast })={T}_{w};\\ {u}^{\ast }(\infty ,{t}^{\ast })=0; & \,{T}^{\ast }(\infty ,{t}^{\ast })={T}_{\infty }.\end{array}$$

There are many theoretical models in the literature which can predict the thermal conductivities of CNTs such as Maxwell, Jeffery, Davis, Hamilton and crosser model. Xue^49^ perceived that the prevailing models are only authentic for spherical or elliptical particles with minor axial ratio. Another, limitation of those models is that they do not deem for the impact of space distribution of CNTs on thermal conductivity. He introduced a theoretical model based on Maxwell theory regarding rotational elliptical nanotubes with very huge axial ratio and compensating the impact of the space distribution on CNTs. Here we use Xue^49^ model for thermal conductivity of CNTs:

$$\frac{{k}_{nf}}{{k}_{f}}=\frac{1-\varphi +2\varphi (\frac{{k}_{CNT}}{{k}_{CNT}-{k}_{f}})\mathrm{ln}\,\frac{{k}_{CNT}+{k}_{f}}{2{k}_{f}}}{1-\varphi +2\varphi (\frac{{k}_{f}}{{k}_{CNT}-{k}_{f}})\mathrm{ln}\,\frac{{k}_{CNT}+{k}_{f}}{2{k}_{f}}}.$$

The density *ρ*_*nf*_, thermal expansion coefficient (*ρβ*)_*nf*_, heat capacitance (*ρc*_*p*_)_*nf*_, are derived by using the relations given by:^50^

$$\begin{array}{rcl}{\rho }_{nf} & = & (1-\varphi ){\rho }_{f}+\varphi {\rho }_{CNT},{(\rho \beta )}_{nf}=(1-\varphi ){(\rho \beta )}_{f}+\varphi {(\rho \beta )}_{CNT}\\ {(\rho {c}_{p})}_{nf} & = & (1-\varphi ){(\rho {c}_{p})}_{f}+\varphi {(\rho {c}_{p})}_{CNT},\end{array}$$

where *ϕ* represents nanoparticles volume fraction, *ρ*_*f*_ and *ρ*_*CNT*_ is the density of the base fluid and CNTs, the volumetric coefficient of thermal expansions of carbon nanotubes and base fluids are denoted by *β*_*CNT*_ and *β*_*f*_ respectively, (*c*_*p*_)_*CNT*_ and (*c*_*p*_)_*f*_ is the specific heat capacities of CNTs and carrier fluids.

Inserting the below non-dimensional quantities:

5 $$u=\frac{{u}^{\ast }}{{U}_{0}},y=\frac{{y}^{\ast }{U}_{0}}{\nu },t=\frac{{t}^{\ast }{U}_{0}^{2}}{\nu },\tau =\frac{\nu {\tau }^{\ast }}{\mu {U}_{0}^{2}},\theta =\frac{{T}^{\ast }-{T}_{\infty }^{\ast }}{{T}_{w}^{\ast }-{T}_{\infty }^{\ast }},$$

into Eqs (1)–(3), we get:

6 $${\varphi }_{2}\frac{{\partial }^{2}u(y,t)}{\partial {y}^{2}}-{\varphi }_{1}(1+\lambda \frac{\partial }{\partial t})\frac{\partial u(y,t)}{\partial t}=-{\varphi }_{3}(1+\lambda \frac{\partial }{\partial t})Gr\theta (y,t),$$

7 $$(1+\lambda \frac{\partial }{\partial t})\tau (y,t)={\varphi }_{2}\frac{\partial u(y,t)}{\partial y},$$

8 $${\varphi }_{5}\frac{{\partial }^{2}\theta (y,t)}{\partial {y}^{2}}-{\varphi }_{4}\Pr \frac{\partial \theta (y,t)}{\partial t}=0,$$

with the corresponding initial and boundary conditions:

9 $$u(y,0)=0,\theta (y,0)=0,y > 0;$$

10 $$u(0,t)=0;\theta (0,t)=1;y > 0,$$

11 $$u(\infty ,t)=0;\theta (\infty ,t)=0,$$

where $$\lambda =\frac{{\lambda }_{1}{{U}^{2}}_{0}}{{\nu }_{f}},Gr=\frac{{\nu }_{f}g{\beta }_{f}{\rm{\Delta }}T}{{{U}^{3}}_{0}},{\rm{\Pr }}=\frac{{(\mu {c}_{p})}_{f}}{{k}_{f}},$$

${U}_{0},\lambda ,Gr,{\rm{\Pr }}$ are the characteristic velocity, Maxwell parameter, Grashoff number and Prandtl number respectively.

$$\begin{array}{rcl}{\varphi }_{1} & = & (1-\varphi )+\varphi (\frac{{\rho }_{CNT}}{{\rho }_{f}}),{\varphi }_{2}=\frac{1}{{(1-\varphi )}^{2.5}},{\varphi }_{3}=(1-\varphi )+\varphi (\frac{{\rho }_{CNT}{\beta }_{CNT}}{{\rho }_{f}{\beta }_{f}}),\\ {\varphi }_{4} & = & (1-\varphi )+\varphi \frac{{(\rho {c}_{p})}_{CNT}}{{(\rho {c}_{p})}_{f}},{\varphi }_{5}=(\frac{1-\varphi +2\varphi (\frac{{k}_{CNT}}{{k}_{CNT}-{k}_{f}})\mathrm{ln}(\frac{{k}_{CNT}+{k}_{f}}{2{k}_{f}})}{1-\varphi +2\varphi (\frac{{k}_{f}}{{k}_{CNT}-{k}_{f}})\mathrm{ln}(\frac{{k}_{CNT}+{k}_{f}}{2{k}_{f}})}),\end{array}.$$

In order to solve the equations (6), (7) and (8), we used the Laplace transform technique and find their solution in the transform (*y*, *q*)- plane.

## Solution of the problem

### Temperature

Exerting Laplace transform of Eqs (8), (10), (11) and using equation (9), emit:

12 $${\varphi }_{5}\frac{{\partial }^{2}\bar{\theta }(y,q)}{\partial {y}^{2}}-{\varphi }_{4}\Pr q\bar{\theta }(y,q)=0,$$

13 $$\bar{\theta }(0,q)=\frac{1}{q},\bar{\theta }(y,q)\to 0\,{\rm{as}}\,y\to \infty .$$

The solution of the partial differential (12) subject to conditions (13) is given as:

14 $$\bar{\theta }(y,q)=\frac{1}{q}\exp (-y\sqrt{{b}_{1}}\sqrt{q}).$$

where $q=\frac{\partial }{\partial t},$ is the Laplace transform parameter, $\bar{\theta }(y,q)=L\{\theta (y,t)\}$ and ${b}_{1}=\frac{{\varphi }_{4}\Pr }{{\varphi }_{5}}.$

Taking the inverse Laplace transform and using (A1), we obtain

15 $$\theta (y,t)=erfc(\frac{y\sqrt{{b}_{1}}}{2\sqrt{t}}).$$

## Velocity field

Taking the Laplace transform of Eqs (6), (10)_1_, (11)_1_ and using initial conditions, we obtain

16 $${\varphi }_{1}(1+\lambda q)q\bar{u}(y,q)={\varphi }_{2}\frac{{\partial }^{2}\bar{u}(y,q)}{\partial {y}^{2}}+{\varphi }_{3}(1+\lambda q)Gr\bar{\theta }(y,q),$$

17 $$\bar{u}(0,q)=0,\bar{u}(y,q)\to 0,\,{\rm{as}}\,y\to \infty .$$

Introducing Eq. (14) into Eq. (16) emits:

18 $$\frac{{\partial }^{2}\bar{u}(y,q)}{\partial {y}^{2}}-{a}_{0}(1+\lambda q)q\bar{u}(y,q)=-{a}_{1}\frac{(1+\lambda q)}{q}\exp (-y\sqrt{{b}_{1}}\sqrt{q}),$$

where $${a}_{0}=\frac{{\varphi }_{1}}{{\varphi }_{2}},{a}_{1}=\frac{{\varphi }_{3}}{{\varphi }_{2}}Gr,$$

Solve the partial differential Eq. (18), we have:

19 $$\bar{u}(y,q)=-{a}_{1}\frac{(\lambda q+1)}{{q}^{2}[{a}_{0}\lambda q+({a}_{0}-{b}_{1})]}\exp (-y\sqrt{{a}_{0}q(\lambda q+1)})+{a}_{1}\frac{(\lambda q+1)}{{q}^{2}[{a}_{0}\lambda q+({a}_{0}-{b}_{1})]}\exp (-y\sqrt{{b}_{1}}\sqrt{q}).$$

The last equality can be written as:

20 $$\begin{array}{rcl}\bar{u}(y,q) & = & -\frac{{a}_{1}}{{a}_{0}\lambda }[\frac{a\lambda -1}{{a}^{2}}\frac{1}{q}+\frac{1}{a}\frac{1}{q}+\frac{1-a\lambda }{{a}^{2}}\frac{1}{q+a}]\exp (-y\sqrt{{a}_{0}q(\lambda q+1)})+\\  &  & +\frac{{a}_{1}}{{a}_{0}\lambda }[\frac{a\lambda -1}{{a}^{2}}\frac{1}{q}+\frac{1}{a}\frac{1}{q}+\frac{1-a\lambda }{{a}^{2}}\frac{1}{q+a}]\exp (-y\sqrt{{b}_{1}}\sqrt{q}),\end{array}$$

where $$a=\frac{{a}_{0}-{b}_{1}}{{a}_{0}\lambda }$$. Let

21 $$F(y,q)=\exp (y\sqrt{\lambda {q}^{2}+q})=\exp (-y\sqrt{\lambda }\sqrt{{(q+\frac{1}{2\lambda })}^{2}-{(\frac{1}{2\lambda })}^{2}}),$$

22 $$\begin{array}{rcl}{\bar{H}}_{1}(y,q) & = & \exp (-y\sqrt{{b}_{1}}\sqrt{q}),\\ {h}_{1}(y,t) & = & {L}^{-1}\{{\bar{H}}_{1}(y,q)\}=\{\begin{array}{c}\frac{y\sqrt{{a}_{0}\lambda }\exp (-\frac{{y}^{2}\lambda {a}_{0}}{4t})}{2t\sqrt{\pi t}};y > 0\\ \delta (t);y=0\end{array}.\end{array}$$

Taking the inverse Laplace transform, Eq. (21) emit:

23 $$\begin{array}{rcl}{f}_{1}(y,t) & = & [{h}_{1}(y,t)+\frac{1}{2\lambda }{\int }_{0}^{t}{h}_{1}(y,z)\frac{z}{\sqrt{{t}^{2}-{z}^{2}}}{I}_{1}(\frac{1}{2\lambda }\sqrt{{t}^{2}-{z}^{2}})dz]\exp (-\frac{1}{2\lambda }t)\\  & = & (\begin{array}{c}\frac{y\sqrt{{a}_{0}\lambda }}{2t\sqrt{\pi t}}\exp (-\frac{{y}^{2}{a}_{0}\lambda }{4t}-\frac{1}{2\lambda }t)\\ +\frac{1}{2\lambda }\exp (-\frac{1}{2\lambda }t){\int }_{0}^{t}\frac{y\sqrt{{a}_{0}\lambda }}{2z\sqrt{\pi z}}\exp (-\frac{{y}^{2}{a}_{0}\lambda }{4z})\frac{z}{\sqrt{{t}^{2}-{z}^{2}}}{I}_{1}(\frac{1}{2\lambda }\sqrt{{t}^{2}-{z}^{2}})dz\end{array}),\end{array}$$

24 $$f(y,t)={L}^{-1}\{F(y,q)\}=\{\begin{array}{c}{f}_{1}(y,t);y > 0\\ \delta (t);y=0\end{array},$$

25 $$G(q)=\frac{a\lambda -1}{{a}^{2}}\frac{1}{q}+\frac{1}{a}\frac{1}{{q}^{2}}+\frac{1-a\lambda }{{a}^{2}}\frac{1}{q+a}.$$

Taking the inverse Laplace, Eq. (25) emits

26 $$g(t)=\frac{a\lambda -1}{{a}^{2}}H(t)+\frac{1}{a}t+\frac{1-a\lambda }{{a}^{2}}\exp (-at),$$

27 $$h(y,t)={L}^{-1}\{\exp (-y\sqrt{{b}_{1}}\sqrt{q})\}=\{\begin{array}{ll}\frac{y\sqrt{{b}_{1}}\exp (-\frac{{y}^{2}{b}_{1}}{4t})}{2t\sqrt{\pi t}} & ;y > 0\\ \delta (t); & ;y=0\end{array},$$

where $\delta (t)$ being Dirac distribution.

Applying the inverse Laplace transform and convolution product, Eq. (20) emits:

28 $$u(y,t)=-\frac{{a}_{1}}{{a}_{0}\lambda }{\int }_{0}^{t}g(t-s)f(y,s)ds+\frac{{a}_{1}}{{a}_{0}\lambda }{\int }_{0}^{t}g(t-s)h(y,s)ds.$$

## Shear stress

Exerting Laplace transform to Eq. (7), emit:

29 $$(1+\lambda q)\bar{\tau }(y,q)={\varphi }_{2}\frac{\partial \bar{u}(y,q)}{\partial y}.$$

Differentiate Eq. (19) with respect to spatial variable $y$, emits

30 $$\begin{array}{rcl}\frac{\partial \bar{u}(y,q)}{\partial y} & = & \frac{{a}_{1}(\lambda q+1)\sqrt{{a}_{0}q(\lambda q+1)}\exp (-y\sqrt{{a}_{0}q(\lambda q+1)})}{{q}^{2}({a}_{0}\lambda q+({a}_{0}-{b}_{1}))}\\  &  & -\frac{{a}_{1}(\lambda q+1)\sqrt{{b}_{1}q}\exp (-y\sqrt{{b}_{1}q})}{{q}^{2}({a}_{0}\lambda q+({a}_{0}-{b}_{1}))},\end{array}$$

Put Eq. (30) into Eq. (29), we obtain:

31 $$\frac{\bar{\tau }(y,q)}{\partial y}=\frac{{a}_{4}(q+{\lambda }_{0})\exp (-y\sqrt{{a}_{0}q(\lambda q+1)})}{q(q+{a}_{2})\sqrt{{a}_{0}q(\lambda q+1)}}-\frac{{a}_{3}\exp (-y\sqrt{{b}_{1}q})}{q(q+{a}_{2})\sqrt{q}},$$

where $${\lambda }_{0}=\frac{1}{\lambda },{a}_{2}=\frac{{a}_{0}-{b}_{1}}{{a}_{0}\lambda },{a}_{3}=\frac{{a}_{1}\sqrt{{b}_{1}}}{{a}_{0}\lambda },{a}_{4}={\varphi }_{2}{a}_{1}.$$

32 $$\bar{\tau }(y,q)=G(q)H(y,q)+g(q)h(y,q),$$

where

33 $$G(q)=\frac{{a}_{4}}{(q+{a}_{2})}+\frac{{\lambda }_{0}{a}_{4}}{q(q+{a}_{2})},$$

34 $$g(q)=\frac{{a}_{3}}{q(q+{a}_{2})}.$$

Using the inverse Laplace transform into Eqs (31), (32), (33) and (34) emits:

35 $$\tau (y,t)={\int }_{0}^{t}{G}_{1}(t-s)\ast {H}_{1}(y,t)ds-{\int }_{0}^{t}{g}_{1}(t-s)\ast {h}_{1}(y,t)ds,$$

with

36 $${G}_{1}(t)={a}_{5}\exp (-{a}_{2}t)+{a}_{6},$$

37 $${g}_{1}(t)={a}_{7}[1-\exp (-{a}_{2}t)],$$

38 $${H}_{1}(t)=\{\begin{array}{ll}0 & ;0 < t < yc\\ {e}^{-{b}_{0}t}{I}_{0}({b}_{0}\sqrt{{t}^{2}-{(yc)}^{2}}) & ;t > yc\end{array}$$

39 $${h}_{1}(t)=\frac{{e}^{-{y}^{2}}\frac{{b}_{1}}{4t}}{\sqrt{\pi t}},$$

## Nusselt number

Nusselt number is $Nu=-\frac{{k}_{nf}}{{k}_{f}}{Re}{\frac{\partial \theta }{\partial y}|}_{y=0},$

40 $$Nu=-\frac{{k}_{nf}}{{k}_{f}}{Re}{(-\frac{\sqrt{{b}_{1}}{e}^{-\frac{{b}_{1}{y}^{2}}{4t}}}{\sqrt{\pi }\sqrt{t}})|}_{y=0}=-\frac{{k}_{nf}}{{k}_{f}}{Re}(-\frac{\sqrt{{b}_{1}}}{\sqrt{\pi }\sqrt{t}}),$$

41 $$Nu=\frac{{k}_{nf}}{{k}_{f}}{Re}\frac{\sqrt{{b}_{1}}}{\sqrt{\pi }\sqrt{t}}=(\frac{1-\varphi +2\varphi (\frac{{k}_{CNT}}{{k}_{CNT}-{k}_{f}})\mathrm{ln}\,\frac{{k}_{CNT}+{k}_{f}}{2{k}_{f}}}{1-\varphi +2\varphi (\frac{{k}_{f}}{{k}_{CNT}-{k}_{f}})\mathrm{ln}\,\frac{{k}_{CNT}+{k}_{f}}{2{k}_{f}}}){Re}\frac{\sqrt{{b}_{1}}}{\sqrt{\pi t}}.$$

where ${R}{{e}}_{x}$ is the Reynold’s number. ${Re}=\frac{x{U}_{0}}{{\nu }_{f}}$.

## Numerical results and discussions

Thermo physical properties of the carrier fluids (Kerosene oil, Engine oil, water and ethylene glycol) and CNTs nanoparticles are given in Table 1. Different values of effective thermal conductivities of CNTs nanofluids are evaluated using Xue model^49^ for various values of volume fraction *ϕ* of CNTs as given in Table 2. It is noticed from these tabulated values that the thermal conductivity is enhanced with elevating volume fraction *ϕ* of CNTs. Also we observed that for the same values of volume fraction *ϕ*, the nanofluids with SWCNTs have higher effective thermal conductivities as compared to that having MWCNTs. The logic for this is the elevated value of thermal conductivity of SWCNTs which is 6600 *Wm* ^−1^/*k* ^−1^ while 3000 *Wm* ^−1^/*k* ^−1^ for MWCNTs as given in Table 1. Nusselt number is computed and numerically the values are calculated to see the effect of nanoparticles’ volume fraction on heat transfer rate. Table 3 shows rate of heat transfer for SWCNT and MWCNT for different values of volume fraction *ϕ*. The enhancement of heat transfer rate can be seen from the table, heat transfer rate increases with increasing volume fraction of nanoparticles which was our main target because the purpose of adding nanoparticles to our working fluid is to enhance its rate of heat transfer and to make it useful for the practical purpose. SWCNTs have higher rate of heat transfer than MWCNTs at the same volume fraction. It is due to the fact that SWCNT has greater thermal conductivity than MWCNT. A comparison of Nusselt number (Nu) which measures the heat transfer rate of the present result with the previous published result of Farhad *et al*.^47^, Table 2 is shown in Table 4. It is found that the present results of Nu for regular fluid (*ϕ* = 0) are in excellent agreement with published results of Farhad *et al*.^47^. This theoretical comparison confirms the accuracy of the present work.

**Table 1:** Thermophysical properties of CNTs and different fluids.

model	*ρ*(*kg*/*m* ^3^)	*c*_*p*_(*kg* ^−1^/*k* ^−1^)	*k*(*Wm* ^−1^ *k* ^−1^)	(*β* × 10^−5^ *k* ^−1^)
water	997	4179	0.613	21
Kerosene oil	783	2090	0.145	99
Engine oil	884	1910	0.144	70
Ethylene glycol	1.115	0.58	0.1490	6.5
SWCNTs	2600	425	6600	27
MWCNTs	1600	796	3000	44

**Table 2:** Thermal conductivity values of CNTs for different values of volume fraction.

Volume fraction *ϕ*	Thermal conductivity *k*_*nf*_ for SWCNT	Thermal conductivity *k*_*nf*_ for MWCNT
0	0.145	0.145
0.01	0.174	0.172
0.02	0.204	0.2
0.03	0.235	0.228
0.04	0.266	0.257

**Table 3:** Heat transfer rate for different values of volume fraction.

Volume fraction *ϕ*	Nusselt number *Nu*_*x*_ for SWCNT	Nusselt number *Nu*_*x*_ for MWCNT
0	1.097	1.097
0.01	1.201	1.194
0.02	1.298	1.284
0.03	1.39	1.371
0.04	1.477	1.453

**Table 4:** Nusselt number Nu (the measure of rate of heat transfer) for the present problem and its comparison with published results of Farhad *et al*.^47^ for regular fluid (*ϕ* = 0).

Pr	*t*	Nu (present result)	Nu (Farhad *et al*.^47^)
0.71	1	0.47	0.47
7	1	1.491	1.492
0.71	2	0.33	0.33

**Figure 4:**
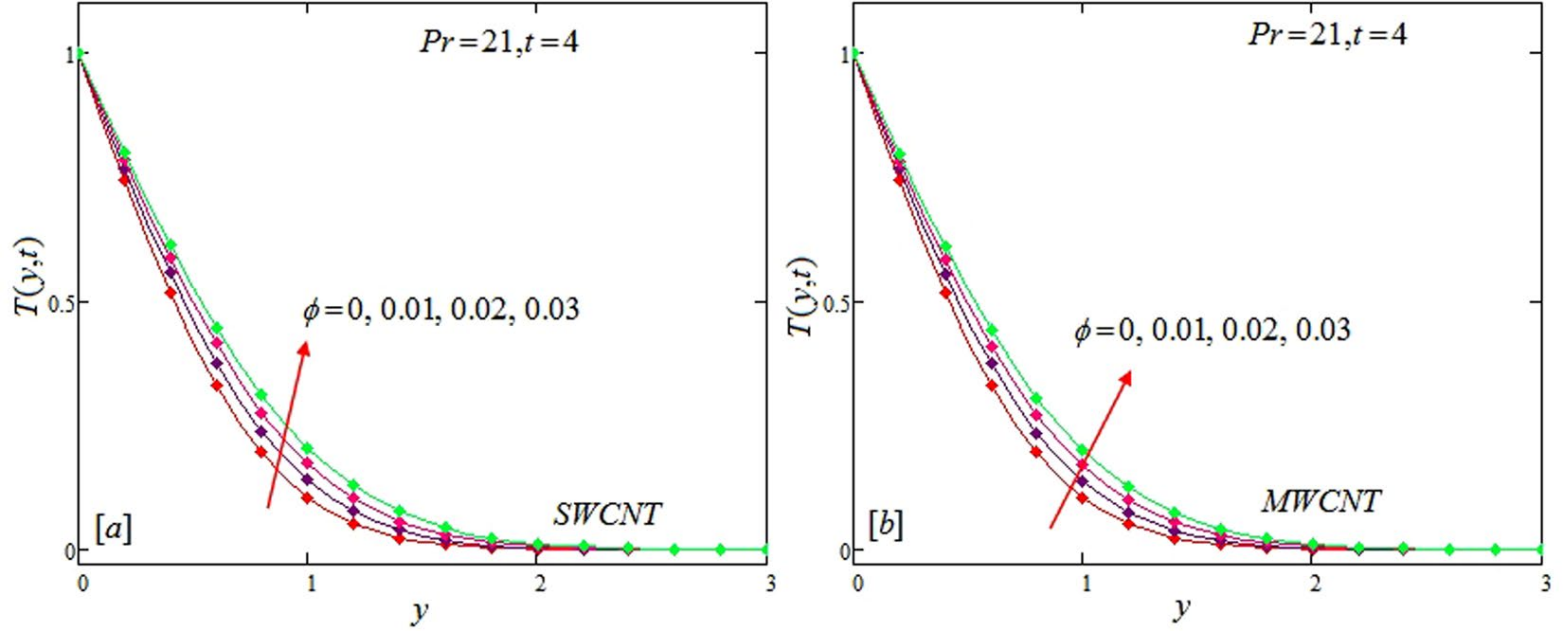
Temperature profiles for single and multiple wall CNTs for different values of volume fraction.

**Figure 5:**
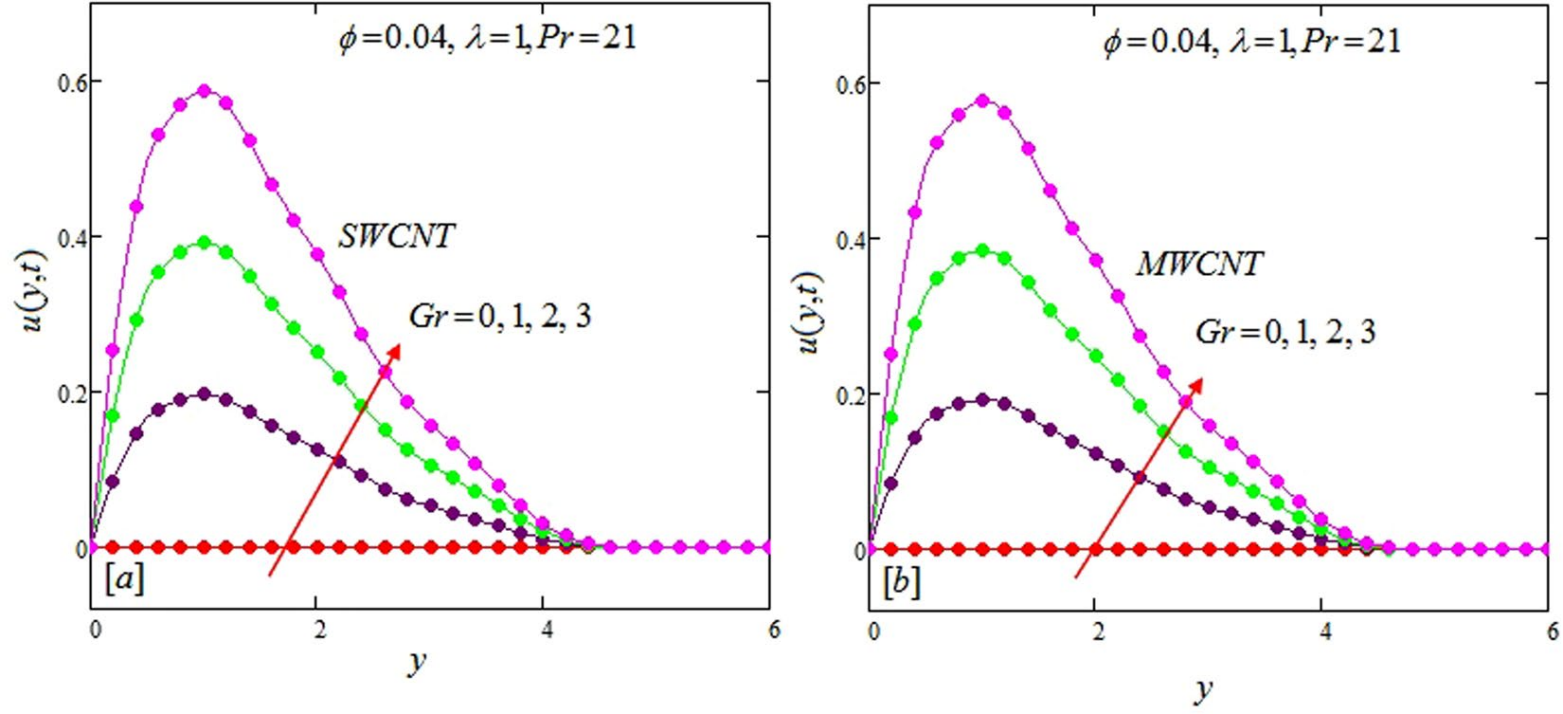
Velocity profiles for single and multiple wall CNTs for different values of Grashoff number.

**Figure 6:**
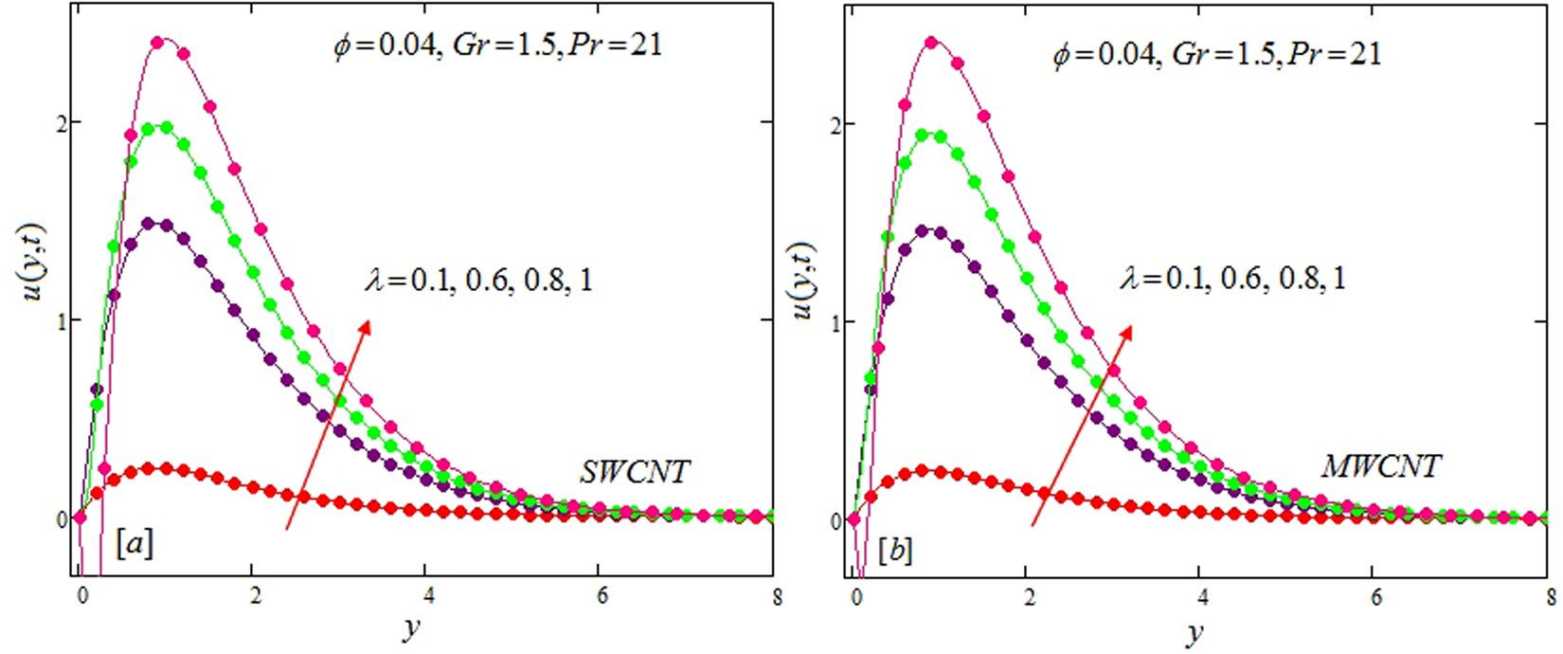
Velocity profiles for single and multiple wall CNTs for different values of Maxwell parameter.

**Figure 7:**
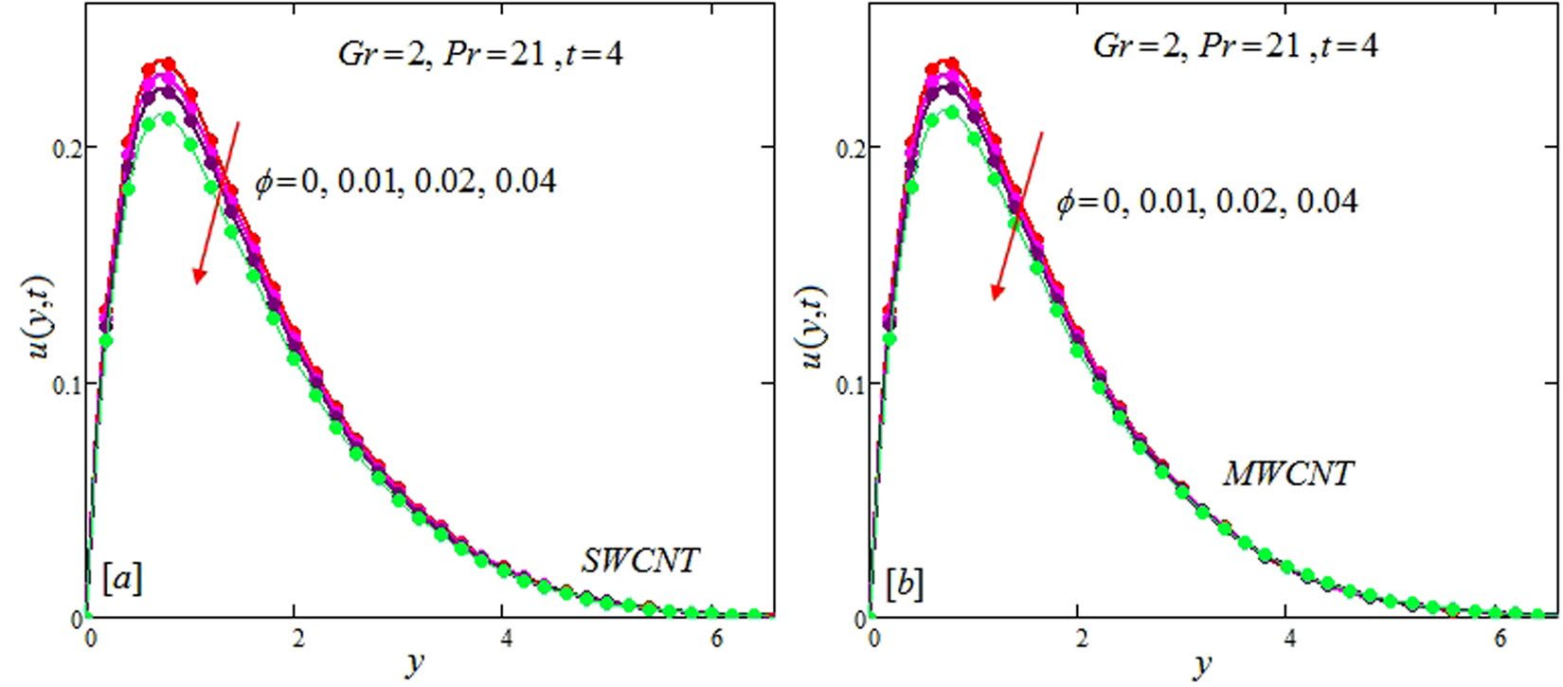
Velocity profiles for single and multiple wall CNTs for different values of volume fraction of CNTs.

**Figure 8:**
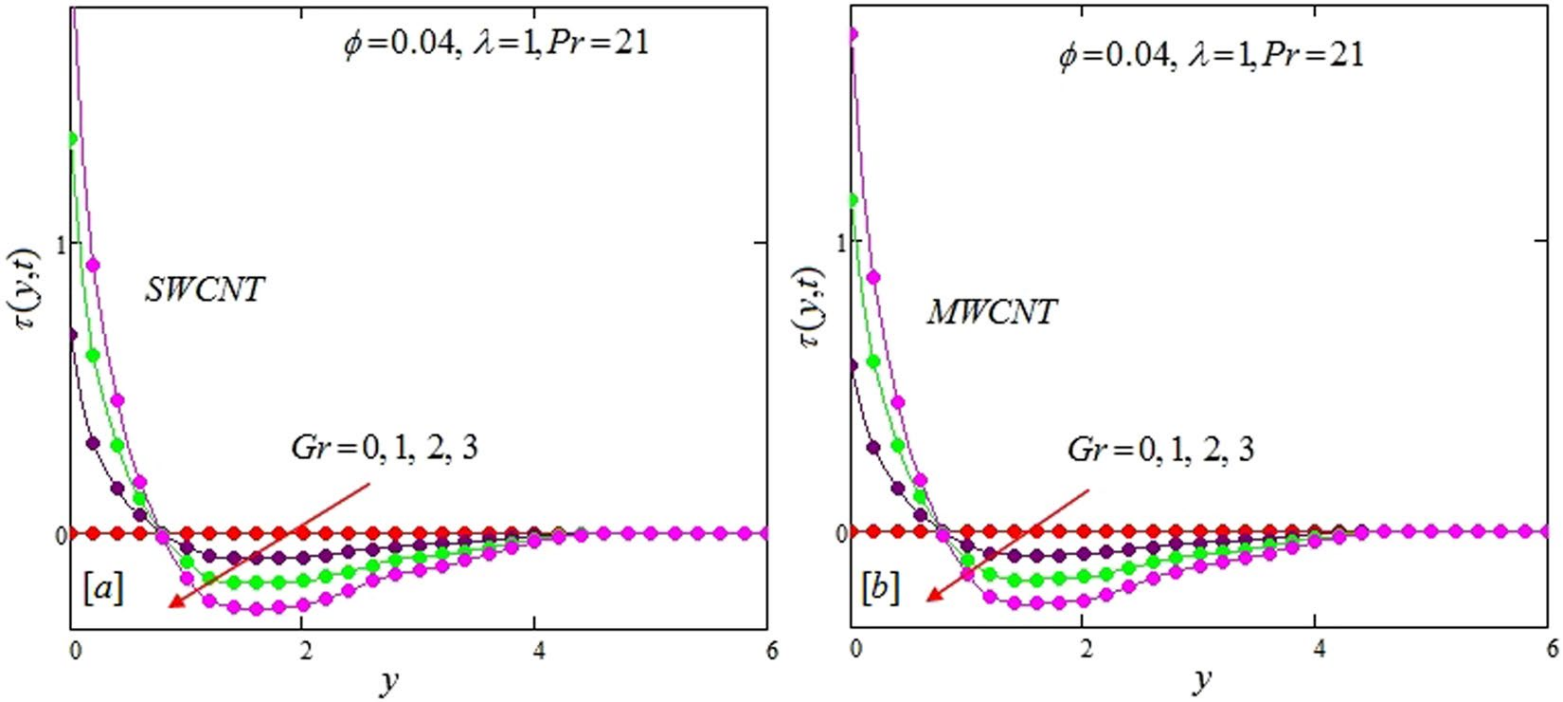
Shear stress profiles for single and multiple wall CNTs for different values of Grashoff number.

**Figure 9:**
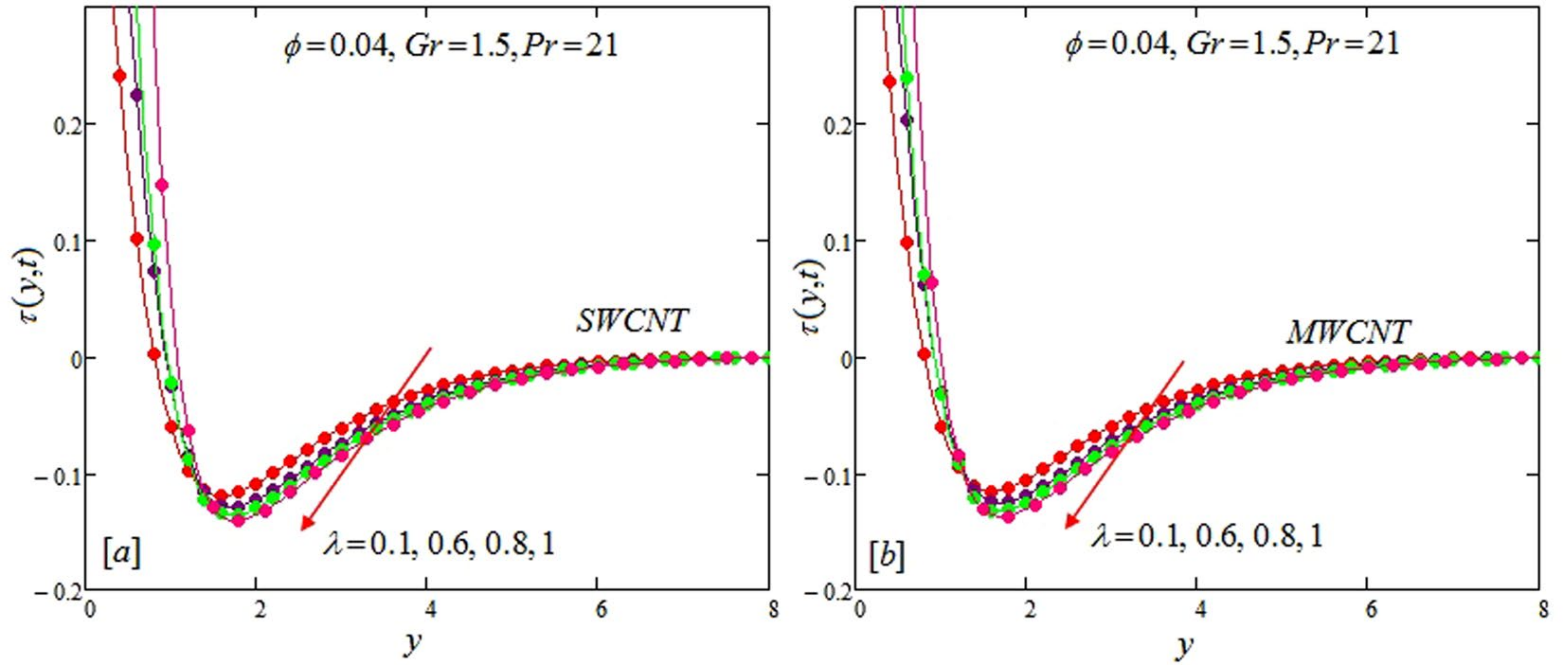
Shear stress profiles for single and multiple wall CNTs for different values of Maxwell parameter.

**Figure 10:**
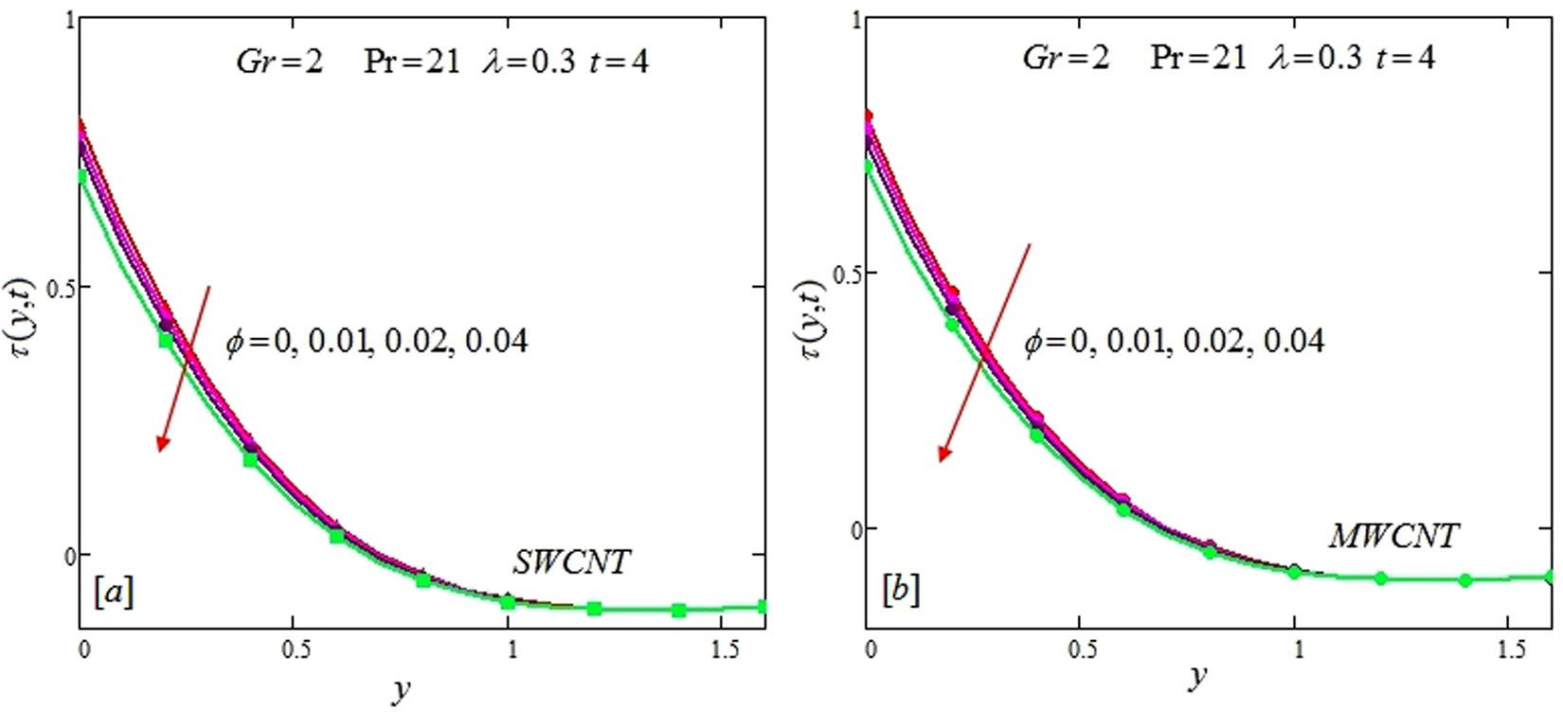
Shear stress profiles for single and multiple wall CNTs for different values of volume fraction of CNTs.

**Figure 11:**
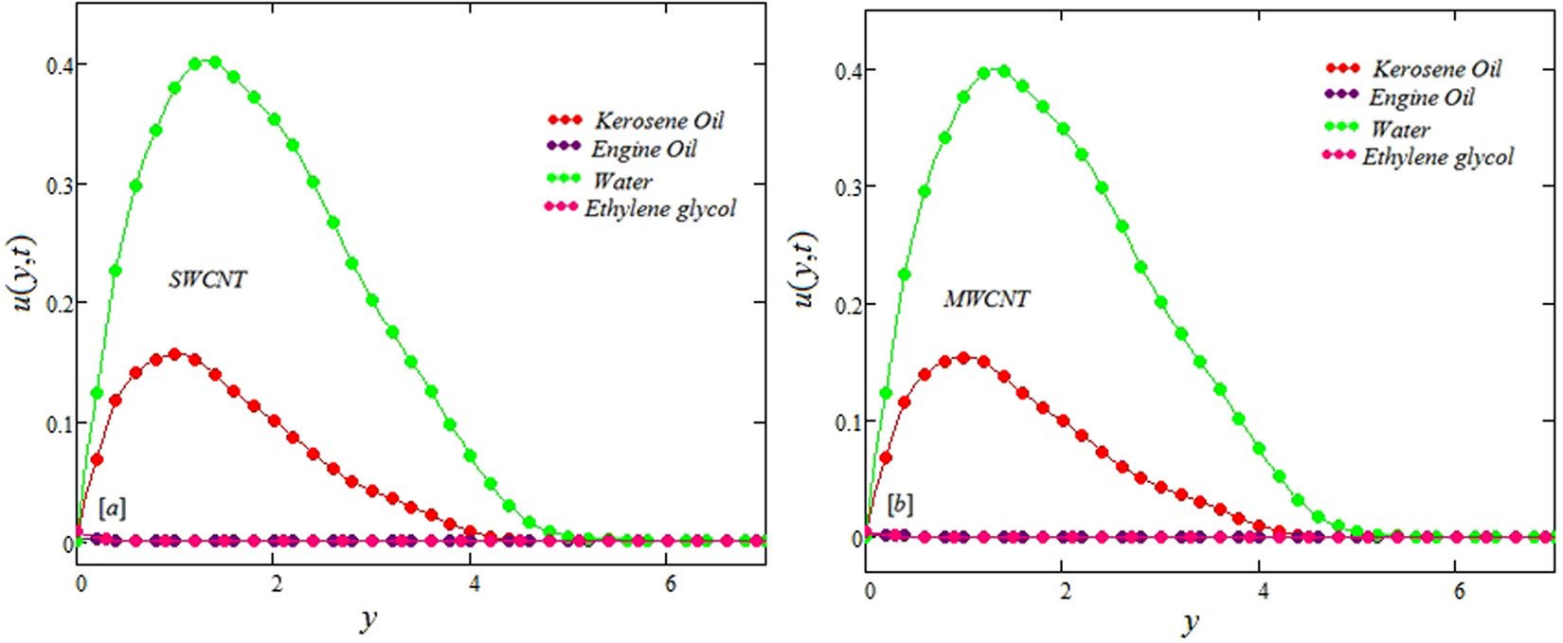
Comparison of velocity profiles for single and multiple wall CNTs for different base fluids.

**Figure 12:**
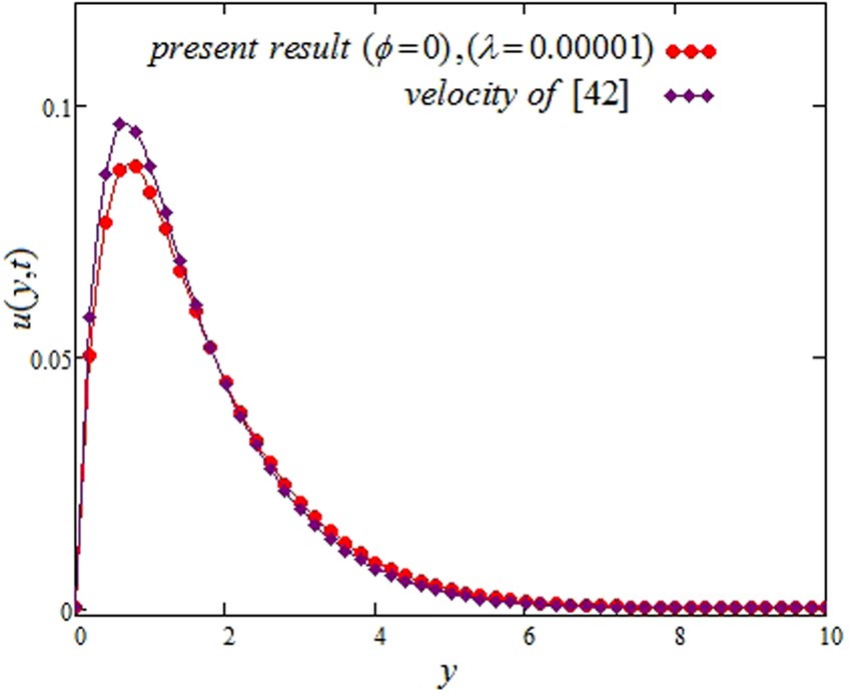
Comparison of velocity profiles for Maxwell and viscous fluids.

The alteration of dimensionless temperature, velocity and shear stress for distinct values of inserted parameter such as Grashof number *Gr*, Maxwell parameter *λ* and nanoparticles volume fraction *ϕ* is studied in Figs 4–12 for Kerosene oil-based Maxwell nanofluid. Also all profiles are plotted versus *y*. Note that in all these figures, (a) and (b) plots shows the results for SWCNTs and MWCNTs respectively. Figure 4(a) and (b) show the alteration of temperature profile with nanoparticles volume fraction *ϕ* of Carbon nanotubes. Temperature is found to be an elevating function of volume fraction *ϕ* for both types of Carbon nanotubes. Physically, the logic is increase in thermal conductivities of nanofluids due to insertion of more CNTs. The more the volume fraction of CNTs in the nanofluids, the more will be its thermal conductivity which in turn enhances the fluids temperature. This increase in thermal conductivities is shown numerically in Table 2. By maximizing the volume fraction *ϕ* of CNTs, a gradual elevation is observed in thermal conductivity. Figure 5(a) and (b) present the velocity profiles for various values of *Gr* for SWCNTs and MWCNTs. It is spotted that the fluid velocity (for both types of CNTs) increases by maximizing the Grashof number *Gr*. When *Gr* = 0, the fluid velocity becomes zero. Physically, it shows that there is no flow in the absence of buoyancy force, as we know that Grashoff number is the ratio of buoyancy force to the viscous force. Thus, here it is concluded from this figure that the flow is due to buoyancy force and in the absence of this force the fluid is in static position. Figure 6(a) and (b) present the impact of Maxwell parameter on the flow of Kerosene-oil based Maxwell nanofluid. It is obvious from these maps that the fluid flow elevates with increasing Maxwell parameter *λ* for both SWCNTs and MWCNTs. Figure 7(a) and (b) show the impact of *ϕ* on the flow of Maxwell nanofluid. It is spotted that velocity profile minimizes with increasing volume fraction *ϕ* for both type of CNTs (SWCNT and MWCNT). Physically, it is due to the fact that the fluids gets viscous by adding nanoparticles and its viscosity/density increases by increasing its amount, thus minimizes the velocity of nanofluid. Figure 8(a) and (b) show the shear stress profiles for SWCNTs and MWCNTs for various values of Grashof number *Gr* It is detected that the shear stress at the bounding wall is zero for *Gr* = 0 For increasing values of Grashof number *Gr* shear stress decreases. Figure 9(a) and (b) present the shear stress profiles for distinct values of Maxwell parameter. The shear stress profile minimizes with increasing values of Maxwell parameter *λ*. Figure 10(a) and (b) show the effect of *ϕ* on profile of shear stress. It is found that shear stress decreases with an elevation of volume fraction *ϕ* of carbon nanotubes (SWCNT and MWCNTs). Comparison graphs are made for SWCNTs and MWCNTs to observe the behavior of nanofluid velocity with various base fluids i.e. Kerosene oil, Engine oil, water and ethylene glycol in Fig. 11(a) and (b). It is clear from these graphs that Maxwell nanofluid with ethylene glycol as base fluid exhibits the lowest velocity. Water-based nanofluids have the highest velocity following by Kerosene oil based nanofluids. Engine oil and Ethylene glycol have lowest velocity profile. Physically the logic is the highest thermal conductivity of water as compared to engine oil, Ethylene glycol and Kerosene oil. Figure 12 shows the comparison of the present results with a previous published result, those of Chandran *et al*.^42^. It is found that at *ϕ* = 0 and *λ* = 0.00001 the present result is in good agreement with the previous result found by Chandran *et al*.^42^, showing that at these values the present Maxwell nanofluid reduces to viscous fluid.

## Conclusions

In this attempt, the first exact solutions for unsteady free convection problem of Maxwell nanofluid are acquired via Laplace transform method. Exact expressions of velocity, shear stress and temperature are acquired and then mapped graphically for various embedded parameters. Carbon nanotubes (SWCNTs and MWCNT) were chosen as nanoparticles inside four different base fluids (Kerosene oil, engine oil, water, ethylene glycol). The impact of volume fraction *ϕ* of CNTs was evaluated and values are obtained by using Xue model^49^ for thermal conductivities as given in Table 2. It is spotted that thermal conductivity has a significant elevation due to maximization in volume fraction *ϕ*. For an increase in Grashof number velocity increases while shear stress were decreased and when *Gr* = 0, they becomes zero which shows graphically that the fluid flow is induced by buoyancy force.

## Electronic supplementary material

Supplementary file
