# Supplementary material for: Heat transfer enhancement in free convection flow of CNTs Maxwell nanofluids with four different types of molecular liquids
Source: Sci Rep. 2017 May 26;7:2445. doi: 10.1038/s41598-017-01358-3 (PMC5446429; doi:10.1038/s41598-017-01358-3)
Supplement: Supplementary file 1 — Supplementary file [file 41598_2017_1358_MOESM1_ESM.doc]

**Heat transfer enhancement in free convection flow of CNTs Maxwell nanofluids with four different types of molecular liquids**

Sidra Aman^1^, Ilyas Khan^2^, Zulkhibri Ismail^3^ and Mohd Zuki Salleh^4^

^1,3,4^Futures and Trends Research Group, Faculty of Industrial Science and Technology, Universiti Malaysia Pahang, Lebuhraya Tun Razak, 26300 UMP Kuantan, Pahang, Malaysia.

*^2^Basic Engineering Sciences Department, College of Engineering Majmaah University, Majmaah 11952, Saudi Arabia.*

^2^Corresponding author email: [ilyaskhanqau@yahoo.com/](mailto:ilyaskhanqau@yahoo.com/) i.said@mu.edu.sa

**Appendix**

$L^{-1}\left\{\frac{exp \left(-c \sqrt{ q+b}\right)}{q- a}\right\}=\frac{exp ( a t )}{2}\left[\begin{matrix} exp\left(-c \sqrt{a + b}\right) erfc\left(\frac{c}{2\sqrt{t}}-\sqrt{( a+ b) t}\right)+ \\ +exp \left( c \sqrt{a + b}\right)erfc \left(\frac{c}{2 \sqrt{t}}+\sqrt{ (a+b)t}\right) \end{matrix}\right].$ (A1)

$L^{-1}\left\{e^{-y\sqrt{a}\sqrt{q}}\right\}=\frac{y\sqrt{a}e^{-\frac{y^{2}a}{4t}}}{2t\sqrt{\pi t}}$ (A2)

$k(t)=L^{-1}\left\{\frac{1}{\sqrt{q}\left(\lambda q+1\right)}\right\}=\frac{1}{\sqrt{\pi\lambda}}\int_{0}^{\infty} \sin\left(\frac{s}{\lambda}\right)\exp\left(-\frac{s^{2}}{4t}\right)ds.$ (A3)

$L^{-1}\left\{\sqrt{q}\exp\left(-a\sqrt{q}\right)\right\}=\frac{a^{2}-2t}{4t^{2}-\sqrt{\pi t}}\exp\left(-\frac{a^{2}}{4t}\right); \mathrm{Re}(a^{2})>0.$ (A4)
